# EEG–Metabolic Coupling and Time Limit at $\dot{V}$O_2_max During Constant-Load Exercise

**DOI:** 10.3390/jfmk10040369

**Published:** 2025-09-26

**Authors:** Luc Poinsard, Christian Berthomier, Michel Clémençon, Marie Brandewinder, Slim Essid, Cécilia Damon, François Rigaud, Alexis Bénichoux, Emmanuel Maby, Lesly Fornoni, Patrick Bouchet, Pascal Van Beers, Bertrand Massot, Patrice Revol, Thomas Creveaux, Christian Collet, Jérémie Mattout, Vincent Pialoux, Véronique Billat

**Affiliations:** 1 Laboratoire Mouvement, Equilibre, Performance et Santé (EA 4445), Université de Pau et des Pays de l’Adour, 65000 Tarbes, France; lpoinsard@univ-pau.fr; 2 Physip, 6 Rue Gobert, 75011 Paris, France; c.berthomier@physip.fr; 3 Laboratoire Interuniversitaire de Biologie de la Motricité (UR 7424), Université Claude Bernard Lyon 1, 69100 Villeurbanne, France; michel.clemencon@univ-rouen.fr (M.C.); thomas.creveaux@gmail.com (T.C.); christian.collet@univ-lyon1.fr (C.C.); vincent.pialoux@univ-lyon1.fr (V.P.); 4 Centre d’Études des Transformations des Activités Physiques et Sportives (UR 3832), Université de Rouen Normandie, F-76000 Rouen, France; 5 Ecole Nationale Supérieure d’Arts et Métiers (ENSAM), 151 Bd de l’Hôpital, 75013 Paris, France; marie.brandewinder@ensam.eu; 6 Institut Telecom Paris, CNRS-LTCI, 91120 Palaiseau, France; slim.essid@telecom-paristech.fr (S.E.); ceciliadamon@gmail.com (C.D.); francois.rigaud@telecom-paristech.fr (F.R.); abenicho@gmail.com (A.B.); 7 Centre de Recherche en Neurosciences de Lyon, Brain Dynamics and Cognition Team, INSERM UMRS 1028, CNRS UMR 5292, Université Claude Bernard Lyon 1, 69100 Villeurbanne, France; manu.maby@inserm.fr (E.M.); lesly.fornoni@inserm.fr (L.F.); patrick.bouchet@inserm.fr (P.B.); jeremie.mattout@inserm.fr (J.M.); 8 Unité Fatigue et Vigilance, Institut de Recherche Biomédicale des Armées (IRBA), 91220 Brétigny-sur-Orge, France; pascal.vanbeers@intradef.gouv.fr; 9 INSA Lyon, Ecole Centrale de Lyon, CNRS, Université Claude Bernard Lyon 1, CPE Lyon, INL, UMR5270, 69621 Villeurbanne, France; bertrand.massot@insa-lyon.fr; 10 Plateforme Mouvement et handicap, Service de Médecine Physique et Réadaptation, Hôpital Henry Gabrielle, Hospices Civils de Lyon, 69230 Saint-Genis-Laval, France; patrice.revol@inserm.fr; 11 Institut Universitaire de France, 75231 Paris, France; 12 Faculté des Sciences du Sport, Université Paris-Saclay, Univ Evry, 91000 Evry-Courcouronnes, France

**Keywords:** electroencephalography, endurance, $\dot{V}$O_2_max, time limit, high-intensity exercise, exhaustion

## Abstract

**Background:** Exercise duration at maximum oxygen uptake ($\dot{V}$O_2_max) appears to be influenced not only by metabolic factors but also by the interplay between brain dynamics and ventilatory regulation. This study examined how cortical activity, assessed via electroencephalography (EEG), relates to performance and acute fatigue regulation during a constant-load cycling test. We hypothesized that oscillatory activity in the theta, alpha, and beta bands would be associated with ventilatory coordination and endurance capacity. **Methods:** Thirty trained participants performed a cycling test to exhaustion at 90% maximal aerobic power. EEG and gas exchange were continuously recorded; ratings of perceived exertion were assessed immediately after exhaustion. **Results:** Beta power was negatively correlated with time spent at $\dot{V}$O_2_max (r = −0.542, *p* = 0.002). Theta and Alpha power alone showed no direct associations with endurance, but EEG–metabolic ratios revealed significant correlations. Specifically, the time to reach $\dot{V}$O_2_max correlated with Alpha/$\dot{V}$O_2_ (*p* < 0.001), Alpha/$\dot{V}$CO_2_ (*p* < 0.001), and Beta/$\dot{V}$CO_2_ (*p* = 0.002). The time spent at $\dot{V}$O_2_max correlated with Theta/$\dot{V}$O_2_ (*p* = 0.002) and Theta/$\dot{V}$CO_2_ (*p* < 0.001). The time-to-exhaustion was correlated with Theta/$\dot{V}$CO_2_ (*p* < 0.001) and Alpha/$\dot{V}$CO_2_ (*p* < 0.001). **Conclusions**: These findings indicate that cortical oscillations were associated with different aspects of acute fatigue regulation. Beta activity was associated with fatigue-related neural strain, whereas Theta and Alpha bands, when normalized to metabolic load, were consistent with a role in ventilatory coordination and motor control. EEG–metabolic ratios may provide exploratory indicators of brain–metabolism interplay during high-intensity exercise and could help guide future brain-body interactions in endurance performance.

## 1. Introduction

Maximal oxygen consumption ($\dot{V}$O_2_max) is widely recognized as a key predictor of cardiorespiratory fitness, reflecting the upper limit of an individual’s ability to uptake and utilize oxygen during high-intensity exercise [1,2]. However, despite its importance, $\dot{V}$O_2_max alone does not fully explain individual differences in endurance performance, especially during high-intensity constant-load efforts [3,4]. Even among athletes with similar aerobic capacities, substantial differences in time to exhaustion (TLIM) have been observed, emphasizing the need to investigate additional physiological and neural determinants of acute fatigue regulation [5,6].

In contrast to incremental tests, high-intensity TLIM protocols offer a unique window into endurance performance by maintaining a fixed workload while allowing physiological and perceptual strain to evolve freely over time. These tests not only reflect $\dot{V}$O_2_ kinetics but also capture the interplay between metabolic stress, neuromuscular adaptation, and central fatigue [7,8,9,10,11]. Among the markers used, TLIM at $\dot{V}$O_2_max and TLIM at 90% of maximal aerobic power (TLIM90%MAP) are particularly valuable, as they assess the capacity to sustain extreme physiological demand [8,9,10]. The latter, adopted in this study, provides a sensitive measure of performance sustainability under high metabolic strain [8,9,12]. Yet, large interindividual differences in TLIM90%MAP remain unexplained, suggesting a major role for central mechanisms, including brain activity, in the regulation of acute fatigue onset [13,14,15].

Beyond $\dot{V}$O_2_, exercise tolerance is heavily influenced by metabolic by-products such as carbon dioxide (CO_2_) and lactate. Specifically, the accumulation of CO_2_ during high-intensity exercise contributes to hypercapnia, stimulating ventilatory control centers and altering perceived exertion [16,17,18]. In this context, elevated arterial CO_2_ pressure activates brainstem chemoreceptors, intensifying respiratory drive and amplifying the sensation of effort, potentially limiting endurance performance [19,20,21]. Furthermore, cerebral oxygenation and CO_2_ regulation influence cortical activation patterns, highlighting the involvement of brain mechanisms in limiting performance [19,22].

Beyond the metabolic and cardiorespiratory limitation of the delay to exhaustion, the Central Governor Model (CGM) posits that the brain continuously integrates afferent feedback from peripheral fatigue signals to adjust motor output and prevent physiological failure [6,23]. In this framework, endurance performance is not solely determined by metabolic exhaustion but also by the central nervous system’s ability to regulate effort perception and sustain voluntary effort [24,25,26]. Supporting this idea, Enders et al. observed increased cortical activity during a TLIM at 85% MAP—close to our protocol—especially in frontal and sensorimotor regions involved in effort regulation [27]. These results suggest the presence of an executive regulatory mechanism consistent with the CGM, modulating effort according to accumulating physiological stress [6,23].

Electroencephalography (EEG) offers a powerful tool to explore such central mechanisms by recording dynamic changes in brain oscillations associated with cognitive demand, motor coordination, and metabolic regulation [27,28,29]. Recent EEG studies emphasize the functional role of oscillations in acute fatigue regulation. Beta oscillations (13–30 Hz), linked to motor control and effort perception, rise with neuromuscular demand and may reflect inhibitory processes preserving efficiency [30,31,32]. Theta (4–7 Hz) and alpha (8–12 Hz) oscillations, involved in cognitive-motor coordination and attentional focus, respectively, may support endurance by facilitating adaptation and reducing interference [33,34,35,36,37,38].

While metabolic parameters such as $\dot{V}$O_2_ and $\dot{V}$CO_2_ provide critical insights into endurance physiology, they do not fully capture the role of central regulation in sustaining effort. Past research has often analyzed EEG and metabolic responses separately [29,39,40,41,42], limiting our understanding of how these systems interact in real time during performance regulation. This gap is particularly relevant in constant-load exercise protocols, where the time to exhaustion—rather than the workload—is the critical endpoint.

Therefore, this study aims to contribute to the improvement of endurance models by integrating $\dot{V}$O_2_ kinetics, the CGM, and neuromuscular fatigue parameters. Specifically, we apply EEG–metabolic power ratios (e.g., Theta/$\dot{V}$O_2_, Alpha/$\dot{V}$O_2_) to capture the interaction between cerebral activity and metabolic demand. Unlike traditional EEG analysis, these ratios reflect the dynamic coupling between brain and body during intense effort.

Previously introduced under incremental protocols [43], we now extend these ratios to a TLIM90%MAP, where duration—rather than workload—is the limiting factor. This approach provides new insights into the temporal interplay between neural regulation and metabolic strain under sustained high-intensity exercise.

By examining how EEG spectral power evolves in relation to ventilatory and metabolic signals during constant-load exercise above the second ventilatory threshold (VT2), we describe associations suggesting that the brain may play a role in the regulation of acute fatigue and endurance capacity under high physiological stress.

We hypothesized that cortical oscillations would show distinct associations with endurance performance under high-intensity exercise. Specifically, beta activity was expected to reflect neural strain related to acute fatigue, whereas theta and alpha bands, when considered in relation to metabolic markers, were expected to be associated with processes supporting ventilatory coordination and sustained performance.

## 2. Materials and Methods

### 2.1. Participants Recruitment and Ethical Approval

Forty-two healthy, physically active young adult males (aged 18–35 years) were initially recruited through posters and direct communication targeting trained sports science students and recreational endurance athletes. Twelve were excluded from the final analysis due to technical or signal quality issues (e.g., excessive EEG artifacts, loss of synchronization, or missing ventilatory segments during the TLIM protocol), resulting in a final sample of 30 participants.

Inclusion criteria were: (1) being non-smokers, (2) engaged in at least 3 h of structured physical activity per week, and (3) free from any known neurological, cardiovascular, or musculoskeletal disorders, as well as from medications that could alter physiological or neural responses. The participants practiced a wide variety of sports, including endurance disciplines (e.g., cycling, trail running, rowing, triathlon), team sports (e.g., football, rugby, water polo), combat sports (e.g., judo, boxing, wrestling), and other activities such as dance or tennis. Although not professional or elite athletes, participants demonstrated good aerobic capacity, with $\dot{V}$O_2_max values ranging from 44 to 75 mL·kg^−1^·min^−1^ (see Table 1), consistent with well-trained recreational profiles [2,3].

All participants provided written informed consent prior to inclusion. The protocol adhered to the ethical standards of the Declaration of Helsinki and received approval from the Léon Bérard Research and Ethics Committee (Approval No. A 13-160).

### 2.2. Experimental Sessions

**Figure 1:**
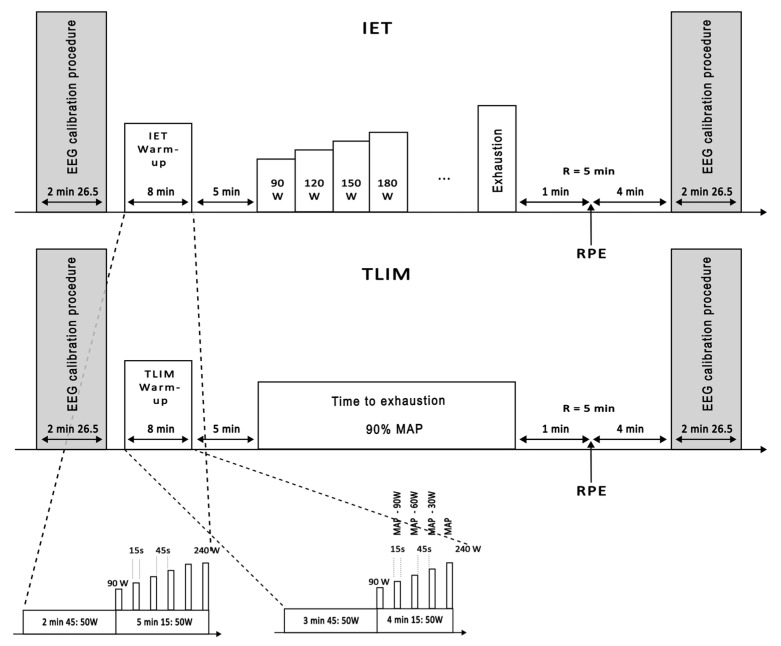
Overview of experimental sessions. Each session began with an EEG calibration procedure lasting approximately 2 min and 26 s, followed by an 8 min warm-up specific to either the Incremental Exercise Test (IET) or the Time-to-Exhaustion Test (TLIM). After a 5 min recovery period, participants performed either the IET, with progressive power increments until exhaustion, or the TLIM, cycling at a constant power corresponding to 90% of maximal aerobic power, sustained until exhaustion. The session concluded with a rate of perceived exertion (RPE) assessment during a 5 min recovery and a final EEG calibration. The warm-up protocols and test procedures are detailed in the figure’s insets. EEG: electroencephalogram; MAP: maximal aerobic power; RPE: rate of perceived exertion.

Each participant completed two laboratory visits separated by 7 to 14 days: (1) an Incremental Exercise Test (IET) and (2) a Time-to-Exhaustion Test (TLIM90%MAP). The first session included anthropometric measurements (age, height, weight, and body mass index). A detailed overview of both sessions is presented in Figure 1.

Each session followed the same general structure: an initial EEG calibration (2 min 26 s), an 8 min warm-up, a 5 min passive recovery, the main exercise protocol (IET or TLIM), a final 5 min recovery with perceived exertion assessment, and a concluding EEG calibration. Specific warm-up protocols and test procedures are illustrated in Figure 1.

#### 2.2.1. Incremental Exercise Test

The IET was designed to determine MAP, $\dot{V}$O_2_max and VT2 [44]. Following the initial EEG calibration, participants completed an 8 min warm-up: 2 min 45 s at 50 W, followed by 5 min 15 s alternating between six 15 s intervals from 90 W to 240 W (in 30 W increments) and 45 s at 50 W. After a 5 min recovery, the IET began at 90 W, with workload increasing by 30 W every 2 min until exhaustion.

- Exhaustion criteria and subjective effort

Participants cycled at a self-selected cadence until volitional exhaustion, defined as the inability to maintain the required power output or a cadence dropping below 60 rpm for more than 10 s, despite three verbal prompts. One minute after exhaustion, they reported their perceived exertion using the Borg scale (6–20), which quantifies subjective effort from ‘no exertion’ to ‘maximal exertion’ [45]. No feedback on time or workload was provided during the test.

- Determination of $\dot{V}$O_2_max, MAP, and VT2

$\dot{V}$O_2_max was identified using a rolling 30 s average, following the recommendations of Niemeyer et al. [46] and Poole et al. [47]. A plateau was considered valid if:

➢ The $\dot{V}$O_2_ increase between two 30 s intervals was <50% of the expected submaximal rise, or
➢ The $\dot{V}$O_2_ increase was ≤150 mL·min^−1^ despite continued workload increase.

If no plateau was observed, $\dot{V}$O_2_max was not considered achieved, in accordance with Poole and Jones [48]. Indeed, these authors emphasize that measuring $\dot{V}$O_2_max based solely on $\dot{V}$O_2_peak is no longer acceptable, as without a clear $\dot{V}$O_2_-work rate plateau, secondary criteria such as respiratory exchange ratio or maximal heart rate may lead to inaccurate assessments.

MAP was defined as the lowest power output at which $\dot{V}$O_2_max was first sustained for at least 30 s, consistent with the operational definition of v$\dot{V}$O_2_max as the minimal velocity eliciting $\dot{V}$O_2_max during incremental protocols [5,7]. This approach extends the concept from treadmill running to cycling power, ensuring that the workload corresponded to an intensity capable of maintaining $\dot{V}$O_2_max over a rolling 30 s window.

VT2, also referred to as the respiratory compensation point, was determined using gas-exchange analysis, marked by a simultaneous rise in $\dot{V}$E/$\dot{V}$O_2_ and $\dot{V}$E/$\dot{V}$CO_2_, along with a decrease in end-tidal CO_2_ pressure [49]. Two trained raters independently identified VT2; a third reviewer resolved any discrepancies.

#### 2.2.2. Time-to-Exhaustion Test

The TLIM test assessed endurance at 90% of MAP, an intensity chosen to ensure the exercise remained within the high-intensity, non-steady-state domain and allowed $\dot{V}$O_2_max attainment before exhaustion [10,50]. After the standard EEG calibration, participants completed an 8 min warm-up adapted from the IET: it included five 15 s ramps up to their MAP, interspersed with 45 s at 50 W. Following a 5 min recovery, participants cycled at 90% MAP until exhaustion.

From this test, three temporal metrics were extracted:

1. TLIM duration: the total time elapsed from the start of the constant-load phase to volitional exhaustion.
2. Time to reach $\dot{V}$O_2_max: the time between exercise onset and the first point at which $\dot{V}$O_2_max was reached, based on plateau criteria.
3. Time spent at $\dot{V}$O_2_max: the remaining time between $\dot{V}$O_2_max attainment and exhaustion, during which $\dot{V}$O_2_ remained at maximal levels.

- Exhaustion criteria and $\dot{V}$O_2_max Confirmation

Exhaustion was defined as either voluntary cessation or inability to maintain ≥30 rpm for 10 s, despite three verbal prompts. $\dot{V}$O_2_max confirmation used the same plateau criteria as the IET [46,47,48]. After task failure, RPE was recorded (mean ± SD = 17.4 ± 1.4), corresponding to ‘very hard’ effort. No temporal or workload feedback was given.

### 2.3. Measurements and Synchronization

#### 2.3.1. Measurements

**Figure 2:**
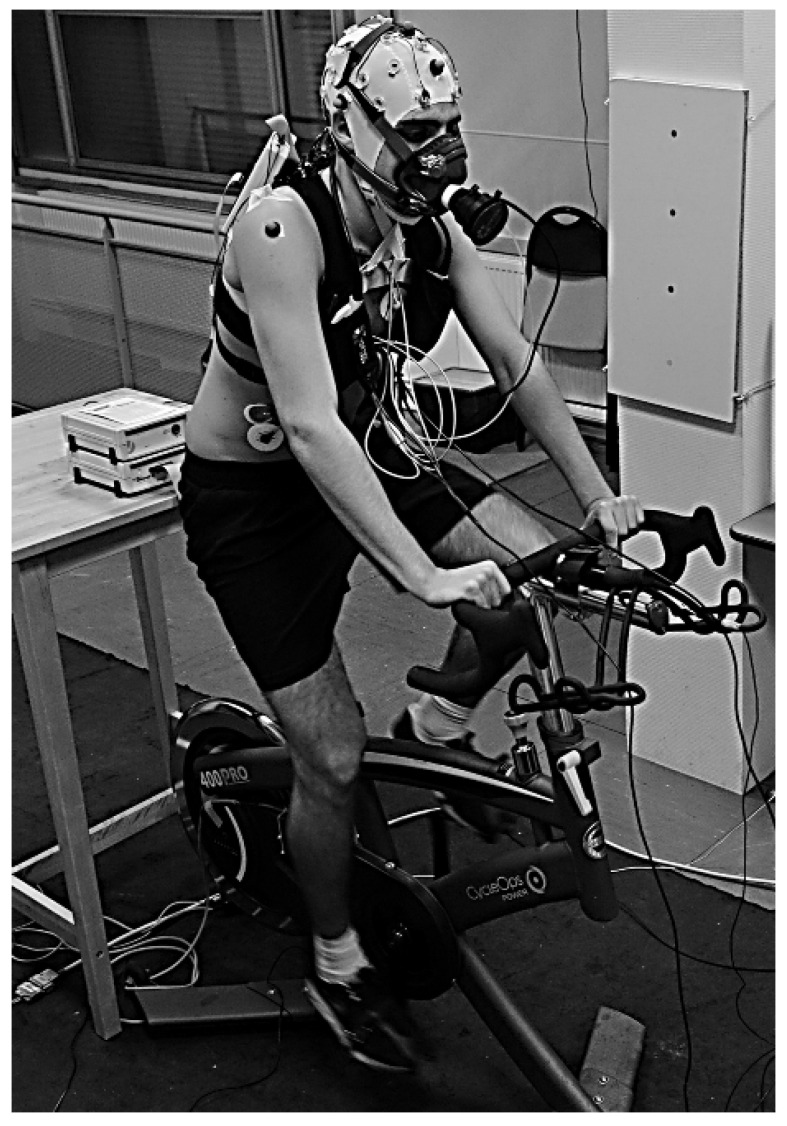
Sensor placement and experimental setup. Reproduced with permission from Billat et al., 2024 [43].

Four synchronized systems were used to record physiological and neurophysiological data during the two test sessions (Figure 2).

1. Cycling parameters: A CycleOps 400 Pro Indoor Cycle (Saris Cycling Group, Inc. 5253 Verona Road Madison WI 53711, USA) measured power output (W), cadence (rpm), torque (Nm), and heart rate (bpm) via ANT+ sensors connected to the Joule 3.0 CPU. Protocols were pre-programmed for consistency across participants. Data was recorded at 1 Hz and analyzed using PowerAgent software (v7.8.28) (Saris Cycling Group, Inc.).
2. Electroencephalography: Brain activity was recorded using a 32-channel ActiCap system (Brain Products, Gilching, Germany), with active gel electrodes placed according to the 10–10 international system; analyses focused on a subset of electrodes (Fp1, Fp2, Fz, C3, Cz, C4, Pz, O1, Oz, O2). To ensure high signal quality and minimize artifacts inherent to intense exercise, a custom-fitted EEG cap was used, with high-quality conductive gel applied to ensure stable contact with the scalp. Impedance was kept <5 kΩ. The reference was placed on the right mastoid and the ground on the lateral third of the spine of the right scapula. We acknowledge that this montage can be susceptible to electromyographic (EMG) or movement artifacts; however, these limitations were specifically addressed during our preprocessing, as detailed in Section 2.4.1. on Artifact Rejection. Signals were amplified (BrainAmp^®^, band-pass 0.016–1000 Hz), sampled at 5000 Hz, then downsampled to 1000 Hz using anti-aliasing filters. Brain Vision Recorder^®^ (v1.20.0601, Brain Products) was used to capture data. Calibration sequences (eyes open/closed, movements) were performed before and after each test.
3. Heart Rate Monitoring: Heart rate (HR) was recorded using two systems. First, a Polar^®^ chest strap synchronized with gas exchange data. Second, a 3-lead electrocardiogram (ECG) with silver electrodes in precordial placement. Instantaneous HR (IHR) was derived from the D2 derivation signal, representing the interval between consecutive R-waves. To address artifacts or missed detections, an offline algorithm corrected IHR variations exceeding a ten beats per minute (bpm) threshold by interpolating between adjacent below-threshold values, producing a clean, resampled signal. The smallest appreciable variation was 0.5 bpm, and the calibrated HR range spanned from 0 to 200 bpm. The IHR time-series was resampled at 10 Hz for subsequent analyses.
4. Gas Exchange: Breath-by-breath $\dot{V}$O_2_ and $\dot{V}$CO_2_, and respiratory exchange ratio (RER) were recorded using a Metamax^®^ 3B analyzer (Cortex Biophysik GmbH, Leipzig, Germany), calibrated before each test according to the manufacturer’s recommendations. Data were synchronized with heart rate recordings and analyzed post-exercise using Metasoft^®^ software (version 3.9.9 SR5, Cortex Biophysik GmbH).

Although the Metamax^®^ 3B system can measure additional ventilatory parameters such as minute ventilation, respiratory frequency, and tidal volume, these variables were not included in the exported dataset and were therefore not analyzed in the present study.

#### 2.3.2. Synchronization

**Figure 3:**
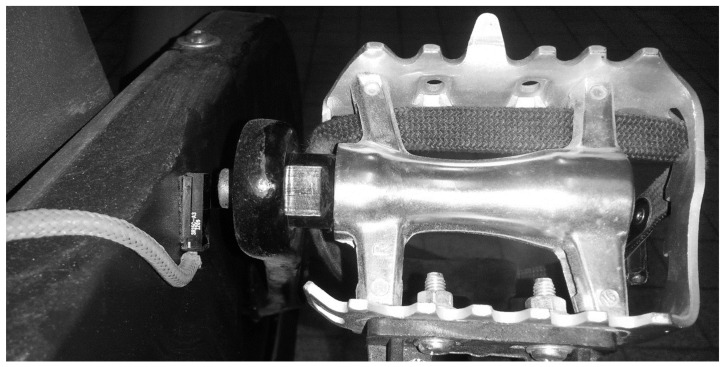
Custom synchronization setup with pedal-mounted magnet and frame-attached Hall sensor. Reproduced with permission from Billat et al., 2024 [43].

Because the ergocycle did not support digital inputs, temporal alignment was ensured using a custom magnetic trigger system (Figure 3). A Hall-effect sensor fixed on the ergometer frame detected a magnet on the crank arm, emitting a pulse at each pedal revolution. This signal was used as a reference to synchronize EEG, ECG, and gas exchange data.

For devices without trigger input (ergometer and gas analyzer), synchronization relied on shared HR signals. The HR recorded by the gas analyzer (via Polar^®^, PolarElectro Oy, FI-90440 Kempele, Finland) was aligned with ECG-derived HR, ensuring accurate temporal matching of all datasets.

### 2.4. EEG Analysis

To ensure high-quality EEG recordings during dynamic exercise, a dedicated preprocessing pipeline was implemented, integrating advanced artifact rejection methods [51] and spectral analysis.

#### 2.4.1. Artifact Rejection

EEG signals were acquired using active gel electrodes with integrated noise reduction circuits. To remove motion- and physiology-related artifacts (e.g., muscle activity, eye blinks) that are prevalent in this context, a multi-step procedure was applied:

1. Initial detection: Sudden signal deviations were flagged using a 2 s sliding window with amplitude thresholding.
2. Advanced filtering: Artifact correction was refined using Non-negative Tensor Factorization (NTF) embedded in a Gaussian source separation framework, as described by Lee et al. [52] and Damon et al. [53]. This method processes EEG signals and auxiliary channels jointly in the time-frequency domain.
3. Auxiliary signals: Eye and muscle activity channels were used to guide the decomposition, optimizing the separation of neural and artifact sources [54,55].
4. Signal reconstruction: Clean EEG signals were reconstructed via Wiener filtering, preserving cerebral oscillations while minimizing distortions [56].

This pipeline maintained a high signal-to-noise ratio (>20 dB at 50 Hz) even during intense cycling efforts, indicating effective attenuation of line-noise. Of the 42 initially recruited participants, 12 (29%) were excluded prior to analysis due to excessive artifacts or desynchronization (see Participants). Because this dataset was processed using a legacy pipeline that did not retain epoch-level rejection flags, precise proportions of rejected epochs or channels could not be extracted. However, no further participants were excluded after preprocessing, and all 30 datasets analyzed met the quality criteria for subsequent spectral analysis.

#### 2.4.2. Spectral Analysis

Following preprocessing, EEG power spectral density (PSD) was computed every 2 s using a Fast Fourier Transform with Hanning window. Analyses focused on three standard frequency bands: Theta (4–7 Hz), Alpha (8–12 Hz), and Beta (13–30 Hz).

Absolute PSD values were calculated for each epoch and electrode of interest.

#### 2.4.3. Slope-Based Indicators

To explore neural adaptations over time during TLIM90%MAP, we analyzed the temporal slopes of EEG activity, rather than instantaneous or absolute values.

Two families of slopes were computed for each participant:

1. EEG PSD Slopes: The evolution of spectral power over the entire duration of the TLIM protocol was quantified using Sen’s slope estimator, a robust, non-parametric method suited for monotonic trends in non-normally distributed data.
2. EEG–Metabolic Ratio Slopes: To assess the coupling between neural and metabolic responses, EEG power was divided by breath-by-breath values of $\dot{V}$O_2_ and $\dot{V}$CO_2_, yielding time series such as Theta/$\dot{V}$O_2_, Alpha/$\dot{V}$CO_2_, or Beta/$\dot{V}$O_2_. Sen’s slope was again applied to these ratio time series to evaluate trend in neuro-metabolic interaction during exercise.

To aid interpretation, representative time courses (e.g., PSD dynamics, %$\dot{V}$O_2_max, EEG–metabolic ratios) from a typical participant are provided in the Supplementary Materials (Figures S1–S3). Note that linear trendlines shown in these figures are for illustrative purposes only and do not correspond to the Sen’s slope values used in statistical analyses.

### 2.5. Statistics

Most statistical analyses were based on non-parametric methods due to the potential non-linearity and non-Gaussian nature of the data. In addition, exploratory regression analyses were performed.

#### 2.5.1. Normality and Justification for Non-Parametric Methods

The distribution of all continuous variables was first assessed using the Shapiro–Wilk test, which indicated significant deviations from normality in most cases (*p* < 0.05). Consequently, non-parametric analyses were adopted throughout the study to ensure robustness against outliers and distributional assumptions.

#### 2.5.2. Correlation Analyses

Relationships between EEG dynamics, ventilatory responses, and endurance performance measures were evaluated using Spearman’s rank correlation coefficient, which does not assume linearity or normal distribution. To account for the risk of Type I errors due to multiple comparisons, the Bonferroni correction was applied to all correlation analyses.

#### 2.5.3. Trend Detection and Slope Estimation

To quantify time-dependent changes during the time-to-exhaustion (TLIM) protocol:

- The Mann–Kendall test was applied to detect monotonic trends in EEG and ventilatory variables across time.
- Sen’s slope estimator was used to calculate the rate of change (slope) for:
  ➢ EEG power (theta, alpha, and beta bands),
  ➢ Ventilatory responses ($\dot{V}$O_2_ and $\dot{V}$CO_2_),
  ➢ EEG–metabolic ratios (e.g., Alpha/$\dot{V}$CO_2_, Beta/$\dot{V}$O_2_).

Sen’s slope estimator was selected for its robustness to outliers, non-linearity, and heteroscedasticity, making it well-suited for small to moderate sample sizes. Unlike parametric linear models, it does not rely on distributional assumptions and provides a resistant estimate of the median trend slope across data pairs. This is particularly relevant given the moderate sample size (n = 30 trained men) and the physiological variability inherent in EEG and ventilatory responses during high-intensity effort. Previous work has highlighted the estimator’s stability and accuracy under these conditions [57,58].

#### 2.5.4. Exploratory Regression Analyses

Exploratory univariate regressions were performed to test whether EEG PSD slopes, EEG–metabolic ratios, and ventilatory slopes predicted endurance outcomes (TLIM duration, time to reach $\dot{V}$O_2_max, and time spent at $\dot{V}$O_2_max). Predictors and outcomes were z-standardized prior to modeling. Results are reported as standardized coefficients (β), standard errors (SE), *p*-values, and model R^2^. Given the small sample size (n = 30), only univariate models were computed to avoid multicollinearity. Multiple-testing control was addressed using a Bonferroni-adjusted significance threshold (α = 0.0033, based on 15 predictors). These regressions are reported as exploratory and complementary to the primary non-parametric analyses.

#### 2.5.5. Focus on Dynamic Adaptation

All statistical analyses involving EEG data, whether spectral power or EEG–metabolic coupling, were conducted exclusively on slope values computed over the full duration of the TLIM test. No statistical test was performed on instantaneous or averaged EEG values. This slope-based approach allowed us to capture dynamic neural and neuro-metabolic adaptations under high-intensity exercise conditions.

#### 2.5.6. Software

Statistical analyses were carried out using XLSTAT (v2024.2.0; Lumivero, Denver, CO, USA).

## 3. Results

Table 2 presents the descriptive statistics of key physiological variables measured during the incremental test. These include indicators of aerobic fitness and exercise intensity, such as $\dot{V}$O_2_max, MAP, maximal HR, and the respiratory exchange ratio.

Turning to the TLIM90%MAP, twenty-three of the thirty participants exhibited a clear $\dot{V}$O_2_max plateau before volitional exhaustion, indicating that the majority stabilized their oxygen uptake at $\dot{V}$O_2_max during the constant-load phase.

Table 3 presents the descriptive statistics of key endurance performance variables measured during the TLIM test, including time-based indicators, perceptual responses, and metabolic markers.

All participants reached a respiratory exchange ratio (RER) above 1.0 at the end of the test, and the average rating of perceived exertion (RPE) was 17.4 ± 1.4, reflecting the high intensity and maximal nature of the effort (Table 3).

To further explore the physiological relevance of maximal oxygen uptake and key endurance performance indicators, we examined the inter-correlations among these metrics. We found that $\dot{V}$O_2_max was not correlated with the time limit at $\dot{V}$O_2_max (r = −0.072, *p* = 0.703), TLIM duration (r = −0.213, *p* = 0.257), or the time to reach $\dot{V}$O_2_max (r = −0.218, *p* = 0.245).

In contrast, we observed significant correlations between the various endurance metrics themselves. TLIM duration was positively correlated with both time to reach $\dot{V}$O_2_max (r = 0.656, *p* < 0.001) and time spent at $\dot{V}$O_2_max (r = 0.786, *p* < 0.001). The correlation between time to reach $\dot{V}$O_2_max and time spent at $\dot{V}$O_2_max was not significant (r = 0.170, *p* = 0.367).

### 3.1. EEG Activity Is Differentially Modulated Across Frequency Bands

Table 4 presents Spearman’s correlation coefficients between EEG power spectral density (PSD) slopes and endurance performance metrics, including TLIM duration, time to reach $\dot{V}$O_2_max, and time spent at $\dot{V}$O_2_max and RPE.

Beta power was negatively correlated with time spent at $\dot{V}$O_2_max (r = −0.542, *p* = 0.002, 95% CI [−0.766, −0.201]). Alpha power showed a positive correlation with Beta power (r = 0.569, *p* = 0.001, 95% CI [0.235, 0.783]). Additionally, RPE was positively correlated with Beta power (r = 0.559, *p* = 0.002, 95% CI [0.222, 0.777]). No other significant correlations were observed between these variables.

### 3.2. Correlations Between the EEG–Metabolic Ratios Dynamics and Time-to-Exhaustion, Time to Reach $\dot{V}$O_2_max or TLIM at $\dot{V}$O_2_max

Table 5 presents Spearman’s correlation coefficients between EEG–metabolic ratios and endurance performance metrics, including TLIM duration, time to reach $\dot{V}$O_2_max, and time spent at $\dot{V}$O_2_max.

The time to reach $\dot{V}$O_2_max was positively correlated with Alpha/$\dot{V}$O_2_ (r = 0.666, *p* < 0.001, 95% CI [0.368, 0.840]) and Alpha/$\dot{V}$CO_2_ (r = 0.722, *p* < 0.001, 95% CI [0.453, 0.871]).

The time spent at $\dot{V}$O_2_max was significantly correlated with Theta/$\dot{V}$O_2_ (r = 0.561, *p* = 0.002, 95% CI [0.224, 0.778]) and Theta/$\dot{V}$CO_2_ (r = 0.635, *p* < 0.001, 95% CI [0.324, 0.822]).

The TLIM duration was correlated with Theta/$\dot{V}$CO_2_ (r = 0.607, *p* < 0.001, 95% CI [0.285, 0.805]) and Alpha/$\dot{V}$CO_2_ (r = 0.611, *p* < 0.001, 95% CI [0.290, 0.808]).

The Beta/$\dot{V}$CO_2_ ratio also showed a significant positive correlation with time to reach $\dot{V}$O_2_max (r = 0.557, *p* = 0.002, 95% CI [0.219, 0.775]). No other significant correlations were observed.

### 3.3. Relationships Between EEG PSD Slopes and $\dot{V}$O_2_ and $\dot{V}$CO_2_ Dynamics During the TLIM Test

Table 6 presents Spearman’s correlation coefficients between EEG power spectral density (PSD), EEG–metabolic ratios, and ventilatory variables ($\dot{V}$O_2_ and $\dot{V}$CO_2_) during the TLIM test.

Beta power was positively correlated with both $\dot{V}$O_2_ (r = 0.677, *p* < 0.001, 95% CI [0.384, 0.846]) and $\dot{V}$CO_2_ (r = 0.557, *p* = 0.002, 95% CI [0.219, 0.775]). No other significant correlations were found.

### 3.4. Regression Analyses of EEG–Metabolic Slopes and Endurance Outcomes

Table 7 presents the exploratory regression models assessing the relationships between EEG PSD slopes, EEG–metabolic ratios, ventilatory slopes, and endurance outcomes.

Before correction, significant associations were observed between Alpha/$\dot{V}$O_2_ slope and time to reach $\dot{V}$O_2_max (*p* = 0.009), Theta/$\dot{V}$CO_2_ slope (*p* = 0.014), Beta/$\dot{V}$CO_2_ slope (*p* = 0.012), $\dot{V}$O_2_ slope (*p* = 0.002), and $\dot{V}$CO_2_ slope (*p* = 0.001) with TLIM duration, as well as $\dot{V}$O_2_ slope (*p* = 0.004) and $\dot{V}$CO_2_ slope (*p* = 0.008) with time spent at $\dot{V}$O_2_max.

After Bonferroni correction, only $\dot{V}$O_2_ and $\dot{V}$CO_2_ slopes remained significantly associated with TLIM duration, while all other associations did not survive correction.

In summary, this study identified key associations between EEG activity, ventilatory responses, perceived exertion, and endurance:

1. Beta power was negatively correlated with time spent at $\dot{V}$O_2_max and positively correlated with both $\dot{V}$O_2_ and $\dot{V}$CO_2_.
2. Alpha power showed a significant positive correlation with Beta power.
3. RPE was positively correlated with Beta power.
4. The time to reach $\dot{V}$O_2_max was positively correlated with Alpha/$\dot{V}$O_2_ and Alpha/$\dot{V}$CO_2_ ratios, while the time spent at $\dot{V}$O_2_max was significantly correlated with Theta/$\dot{V}$O_2_ and Theta/$\dot{V}$CO_2_ ratios.
5. TLIM duration was correlated with Theta/$\dot{V}$CO_2_ and Alpha/$\dot{V}$CO_2_ ratios.
6. The Beta/$\dot{V}$CO_2_ ratio was also found to be positively correlated with time to reach $\dot{V}$O_2_max.
7. Exploratory regression analyses showed that slopes of $\dot{V}$O_2_ and $\dot{V}$CO_2_ were significantly associated with TLIM duration after Bonferroni correction. No other associations remained significant after correction.

## 4. Discussion

The overarching objective of this study was to refine endurance physiology models by integrating three key components: (1) gas exchange kinetics ($\dot{V}$O_2_, $\dot{V}$CO_2_), (2) neuromuscular fatigue mechanisms, and (3) central regulatory processes as indexed by EEG activity. By investigating EEG–metabolic interactions during a constant-load time-to-exhaustion protocol, we aimed to better understand the associative relationships between brain activity and endurance limitation under high physiological stress.

Our findings reveal that specific EEG indices are robustly associated with endurance performance and physiological responses during high-intensity exercise. These neurophysiological dynamics show significant correlations with specific endurance and metabolic metrics, suggesting that brain oscillations are linked with sustained effort, but without allowing for causal inference. They should therefore be interpreted as associations that may reflect, rather than directly regulate, fatigue processes.

Compared to our previous studies using self-paced or incremental protocols [43,60], the present experiment provides a more integrative model by incorporating EEG-to-metabolic power ratios and time-to-exhaustion data. This approach captures not only the brain’s activity under load, but also its statistical relationship with metabolic demands, thereby offering fresh insights into the potential associations between central processes and endurance performance during sustained high-intensity exercise.

### 4.1. EEG Activity Differentially Modulated Across Frequency Bands

#### 4.1.1. Beta Power and Endurance

Our findings reveal a significant negative correlation between beta power and time spent at $\dot{V}$O_2_max. This indicates that increased beta activity is linked with greater cortical demand and shorter tolerance at maximal aerobic capacity. Unlike incremental protocols—where workload increases gradually and pacing adjustments can occur—the TLIM protocol used here requires subjects to sustain a fixed, near-maximal effort until exhaustion. This constant-load format may exacerbate the involvement of cortical motor circuits, thereby amplifying the relevance of beta oscillations in endurance regulation [61].

Beta oscillations have been consistently associated with neuromuscular control and motor execution under conditions of increasing metabolic stress [29,30,41,62,63]. In high-intensity constant-load exercise, sustained effort necessitates continuous motor unit recruitment and compensatory mechanisms to counter fatigue-induced force declines [25,64,65,66]. Accordingly, elevated beta power may reflect not only fatigue accumulation but also an adaptive cortical response aimed at sustaining performance despite rising physiological strain. Furthermore, the high metabolic demand inherent to exercise in the high-intensity domain—particularly around 90%MAP—is known to drastically shorten time to exhaustion due to rapid energy depletion and impaired homeostasis [67,68]. The negative association between beta power and time spent at $\dot{V}$O_2_max may therefore indicate the neurophysiological cost of sustaining neuromuscular drive under extreme metabolic constraints, rather than proving a direct causal mechanism.

#### 4.1.2. Alpha Power and Endurance Maintenance

Our analyses did not reveal a statistically significant correlation between alpha power and any endurance metrics after Bonferroni correction. However, the observed tendency for a negative relationship between alpha power and time spent at $\dot{V}$O_2_max (*p* = 0.006) warrants discussion within the existing literature, especially in light of the central role this metric plays in overall endurance performance.

Alpha rhythms are classically linked to sensory processing, attentional regulation, and motor planning. Lower alpha power is generally interpreted as a marker of increased cortical excitability [64,65,69]. Conversely, the increased alpha power observed in individuals with shorter $\dot{V}$O_2_max durations may indicate disengagement or diminished cortical drive, which could be interpreted as a protective neural mechanism during extreme fatigue.

The literature remains equivocal regarding the role of alpha activity in endurance. While some studies suggest that increased alpha power supports efficient sensory gating and energy conservation during repetitive tasks [36,38,70], others report that elevated alpha during fatigue correlates with decreased motor output and attentional withdrawal [35,71]. Our findings, while not statistically conclusive, appear more consistent with the latter interpretation, suggesting that alpha augmentation may be associated with performance limitations by signaling cortical withdrawal or suboptimal neuromotor coordination as perceived effort rises.

#### 4.1.3. EEG–Metabolic Interactions and Acute Fatigue Regulation

A key novelty of our study lies in the exploration of how EEG spectral power correlates with metabolic variables. Following Bonferroni correction, we found that beta power was significantly associated with $\dot{V}$O_2_ and $\dot{V}$CO_2_, whereas no significant correlations were observed for alpha and theta power. These findings suggest that cortical activity, particularly in the beta band, is linked with increased neuromuscular and ventilatory demands during sustained exercise, and appear not to be related to the initial kinetics of oxygen uptake.

Beta power, as noted earlier, is closely tied to neuromuscular coordination, consistent with its proposed role in motor output regulation and corticospinal involvement during sustained effort [61,69]. Its significant association with higher $\dot{V}$O_2_ and $\dot{V}$CO_2_ levels further reinforces the idea that increased cortical motor involvement may be associated with the ability to maintain motor output in the face of escalating metabolic stress [29,30,31,62]. In contrast, the lack of significant correlation for alpha and theta power with metabolic variables may be attributed to the nature of the TLIM protocol. While alpha and theta rhythms are generally linked to attentional regulation and pacing decision [33,35,39,70,71,72,73], they may be less critical to sustaining effort in our fixed-intensity protocol.

These results support the idea that cortical contributions to endurance are dynamic and intensity-dependent. Importantly, they do not establish direct modulation of the time to reach $\dot{V}$O_2_max, rather, they are consistent with sustained neuromuscular coordination and motor output once $\dot{V}$O_2_max is achieved [74,75]. To better capture the complexity of these interactions, we introduced EEG–metabolic power ratios—novel indices that integrate brain activity and systemic metabolism (e.g., Theta/$\dot{V}$O_2_, Alpha/$\dot{V}$O_2_) [43].

### 4.2. The Interplay Between Metabolic and Neurophysiological Power During Endurance Exercise

#### 4.2.1. EEG–Metabolic Ratios and Endurance Performance

Our results revealed that specific EEG–metabolic ratios were associated with endurance performance. These findings offer new insights into the potential relationships between neural and metabolic systems under high-intensity conditions. While most previous studies have analyzed EEG and metabolic responses in isolation [29,39,40,41], our approach using EEG–metabolic ratios provides an integrated framework to examine statistical associations between brain activity and systemic physiology during acute fatigue. This multidimensional approach highlights the added value of normalizing EEG activity to metabolic markers, allowing us to distinguish between oscillations that reflect absolute neural activation and those that may be more closely related to the efficiency of supporting metabolic demands.

#### 4.2.2. Beta Oscillations: Neuromuscular Compensation Rather than Metabolic Efficiency

Although Beta power alone was linked with endurance limitations, its normalization to $\dot{V}$O_2_ (Beta/$\dot{V}$O_2_) did not correlate significantly with performance metrics. Interestingly, the Beta/$\dot{V}$CO_2_ was positively correlated with time to reach $\dot{V}$O_2_max. This pattern suggests that Beta oscillations are more likely linked with neuromuscular and ventilatory compensation strategies than with oxygen utilization efficiency.

Beta-band activity has been repeatedly associated with corticospinal drive and motor coordination under demanding conditions [30,31,69]. Given that CO_2_ production rises with metabolic stress and is tightly linked to ventilatory compensation [76,77], the Beta/$\dot{V}$CO_2_ ratio may reflect increased cortical involvement in sustaining motor and ventilatory functions under strain. This interpretation is consistent with studies reporting elevated Beta activity during tasks requiring precise motor control under fatigue [27,28,29].

The absence of a significant Beta/$\dot{V}$O_2_ association is consistent with the interpretation that Beta activity is more closely related to central adjustments than peripheral metabolic efficiency. In summary, Beta oscillations—particularly when expressed relative to $\dot{V}$CO_2_—may be indicative of compensatory processes in the face of rising neuromuscular and ventilatory demands, rather than serving as a straightforward indicator of endurance capacity.

#### 4.2.3. Theta Oscillations: Autonomic Integration and Ventilatory Efficiency

Unlike Beta activity, Theta power alone was not significantly related to endurance variables. However, both Theta/$\dot{V}$O_2_ and Theta/$\dot{V}$CO_2_ were positively associated with time spent at $\dot{V}$O_2_max. Furthermore, the Theta/$\dot{V}$CO_2_ ratio was positively correlated with TLIM duration. This pattern suggests that Theta activity is associated with endurance outcomes through mechanisms that go beyond raw metabolic output.

Theta oscillations have been linked to autonomic control, interoception, and sensorimotor integration [33,34]. Their positive association with endurance performance metrics may reflect closer coordination between central regulation and peripheral demand. In this sense, higher Theta power relative to metabolic load might indicate a more efficient integration of breathing regulation with neuromuscular demands, potentially supporting prolonged performance without increasing ventilatory cost.

Our findings align with literature reporting associations between Theta activity and breathing patterns or cardio-respiratory coherence, particularly under high cognitive or physical demands [72,73]. In high-intensity fixed-load efforts, where pacing is constrained, such associations may be consistent with stabilizing internal physiology and delay exhaustion.

Thus, Theta oscillations—especially when expressed in relation to metabolic markers—may be indicative of central efficiency, being statistically associated with stabilized physiological responses rather than amplified metabolic power output.

#### 4.2.4. Alpha Oscillations: Balancing Attention, Motor Efficiency, and Metabolic Strain

Alpha activity power alone was not directly associated with TLIM duration. However, when normalized to $\dot{V}$O_2_ and $\dot{V}$CO_2_, Alpha/$\dot{V}$O_2_ and Alpha/$\dot{V}$CO_2_ ratios showed significant positive correlations with time to reach $\dot{V}$O_2_max. Additionally, Alpha/$\dot{V}$CO_2_ was also significantly correlated with TLIM duration. These results are consistent with the idea that Alpha oscillations may be involved in effort regulation through dual associations depending on context.

On one hand, increased Alpha power may reflect disengagement or reduced voluntary motor drive under fatigue [29,70], which aligns with its negative association when considered alone. On the other hand, when expressed relative to metabolic cost, Alpha oscillations may reflect a strategy of neural economy—regulating attentional focus, sensory gating, and motor efficiency [35,38,71]. By minimizing unnecessary cortical activation, Alpha activity could be associated with more efficient cognitive-motor coordination, particularly under conditions of metabolic constraint.

This modulation of neural demand may be associated with task execution and prevention of excessive cognitive fatigue. Therefore, Alpha oscillations do not appear to act as direct drivers of endurance performance but rather as markers of central resource allocation, with their associations suggesting regulation of cortical excitability in proportion to physiological strain.

#### 4.2.5. Perceived Exertion and Its Partial Dissociation from Endurance Regulation

Our findings revealed that RPE was significantly correlated with Beta power but not with Alpha or Theta activity, nor with $\dot{V}$O_2_, $\dot{V}$CO_2_ or any direct endurance performance metrics. These results highlight a partial dissociation between subjective perception of effort and objective performance outcomes. The association between Beta activity and RPE is consistent with the notion that cortical motor engagement is linked with perceived exertion, particularly under high neuromuscular demand. This aligns with previous studies indicating that Beta oscillations reflect increased corticospinal drive and motor coordination effort during fatiguing tasks [27,29,30].

However, the absence of correlation between RPE and performance metrics (e.g., TLIM, time to $\dot{V}$O_2_max, time at $\dot{V}$O_2_max) suggests that perceived exertion does not directly predict endurance capacity under high-intensity, constant-load conditions. While RPE captures an important aspect of exercise experience, it may not fully reflect the underlying neuromuscular and metabolic mechanisms that determine performance in such contexts [78,79].

Interestingly, the lack of direct association between absolute Alpha and Theta power and RPE may indicate that attentional or autonomic modulations—typically attributed to these frequencies—do not systematically influence conscious effort perception. However, our findings suggest that Theta activity relative to metabolic output (e.g., Theta/$\dot{V}$CO_2_) is associated with endurance outcomes, possibly reflecting unconscious integration of interoceptive and ventilatory feedback.

Together, these findings suggest that RPE represents a conscious integrative signal of neuromuscular and metabolic stress but remains insufficient on its own to explain performance outcomes during high-intensity exercise. Furthermore, this apparent dissociation should be interpreted with caution, as the constant-load TLIM protocol may lead to RPE saturation, potentially masking a true relationship between perceived effort and performance during the final stages of the exercise.

#### 4.2.6. Exploratory Regression Analyses

Exploratory regression models were conducted to test whether EEG PSD slopes, EEG–metabolic ratios, and ventilatory slopes were associated with endurance outcomes. Several predictors showed uncorrected associations consistent with the correlation analyses, particularly Beta slopes and EEG–metabolic ratios with TLIM duration and $\dot{V}$O_2_max-related measures. Notably, ventilatory slopes of $\dot{V}$O_2_ and $\dot{V}$CO_2_ significantly predicted TLIM duration even after Bonferroni correction. No EEG or EEG–metabolic predictors survived correction.

This pattern suggests that ventilatory dynamics show robust associations with endurance under severe exercise, whereas EEG and EEG–metabolic indices, although correlated with performance outcomes, did not remain significant once stringent multiple-testing control was applied. The discrepancy between correlation and regression findings for EEG variables may be explained by the modest sample size (n = 30), which limits power, as well as the univariate nature of the regressions, which cannot account for potential collinearity and interdependence among neural and ventilatory signals. Furthermore, the conservativeness of Bonferroni correction increases the risk of Type II errors in exploratory studies with small samples.

Taken together, these findings indicate that ventilatory slopes are strongly linked to endurance outcomes, while EEG and EEG–metabolic slopes may capture additional but more subtle aspects of neuro-metabolic integration that require larger multivariate designs to be fully clarified.

### 4.3. Practical Implications

Taken together, our findings indicate that endurance performance appears to be influenced not only by peripheral metabolic supply but also by central neural mechanisms involved in motor control, ventilatory adaptation, and perceived exertion. The observed associations between EEG–metabolic ratios and endurance measures are consistent with the relevance of brain–body coordination in sustaining effort during high-intensity exercise. Specifically, Beta oscillations were associated with compensatory motor and ventilatory demands, Theta with autonomic and ventilatory integration, and Alpha with cognitive-motor coordination under stress.

These neurophysiological markers open new avenues for understanding how the brain interacts with endurance, but they also raise practical questions. From a performance perspective, approaches aimed at enhancing brain–body communication and reducing unnecessary cortical activity have been proposed as potential training strategies [80,81]. Methods such as attentional control, mindfulness, or neurofeedback training might influence acute fatigue regulation by modulating cortical involvement in effort and ventilation [82,83]. However, these approaches remain experimental and require further validation in athletic populations.

In real-world settings, EEG-based monitoring remains limited due to motion artifacts, sweat interference, and technical constraints [84,85,86]. As such, its practical application in field conditions is currently restricted. In contrast, the Rating of Perceived Exertion offers a simple, validated, and ecologically valid alternative. Although our study found a significant association between RPE and Beta power, RPE remains a widely used tool for pacing, integrating both central (neural) and peripheral (metabolic) feedback in real time [87,88,89].

Notably, recent findings demonstrated that an incremental protocol regulated by RPE (self-paced) elicited EEG and physiological responses comparable to those of a traditional power-driven incremental test [60]. This is consistent with the notion that RPE appears to capture an integrated signal encompassing both cortical and metabolic demands, reinforcing its validity as a proxy for internal workload regulation. RPE has also been reported to guide intensity regulation more effectively than isolated physiological markers, especially in dynamic environments [90,91]. Unlike EEG, it does not require external instrumentation and can be reliably implemented across various endurance disciplines. Thus, while EEG-derived insights contribute to the theoretical understanding of acute fatigue regulation, RPE remains the most accessible and practical tool for optimizing pacing strategies in both recreational and competitive settings.

Accordingly, practical applications of EEG remain preliminary, and any brain-centered training or monitoring should be considered exploratory until validated in larger, diverse cohorts and under improved wearable-EEG conditions.

### 4.4. Study Limitations

While this study offers novel insights into the neural regulation of endurance performance, several limitations must be considered. First, EEG acquisition during high-intensity cycling remains technically challenging. Despite rigorous artifact rejection procedures, motion- and sweat-related artifacts cannot be entirely avoided, which may affect signal quality. Future research should critically evaluate the signal quality and reliability of portable EEG systems under dynamic exercise conditions [92,93] and consider the integration of EEG with other non-invasive techniques such as near-infrared spectroscopy or surface electromyography. These combined approaches could improve cortical signal discrimination, reduce peripheral contamination, and offer a more comprehensive view of cerebrovascular and neuromuscular dynamics during real-world endurance tasks [94].

Second, the EEG–metabolic ratios proposed in this study introduce a novel perspective on brain–body interactions. However, their physiological interpretation and external validity require further clarification. Because EEG power and metabolic measures differ in units and scales, such ratios are sensitive to denominator noise and prone to mathematical coupling, which can artificially inflate associations. Therefore, these indices should be considered as heuristic normalization approaches and should not be interpreted as direct markers of physiological efficiency. It remains to be determined whether these ratios can reliably predict endurance performance across diverse exercise modalities, intensities, or populations—or if they primarily reflect compensatory neural strategies under metabolic stress. Longitudinal and interventional studies are needed to assess their sensitivity to training adaptations and their potential as biomarkers of endurance capacity.

Third, our sample included only healthy, physically active young men; therefore, generalizability to women, older adults, or clinical populations is limited. Although the current sample size was adequate to detect moderate-to-large effect sizes in correlational analyses, future investigations should include larger and more heterogeneous cohorts. Inter-individual differences in EEG–metabolic coupling may be influenced by factors such as aerobic fitness, training background, and psychological characteristics (e.g., interoceptive awareness, motivational traits, attentional control). Addressing these sources of variability is essential to better understand the neural basis of endurance and to develop personalized, brain-informed strategies for fatigue management.

### 4.5. Future Research Directions

As EEG research in time-to-exhaustion protocols remains scarce, our findings call for broader investigations to expand the understanding of brain contributions to endurance regulation. Future studies could consider the following directions:

- Integrative neurophysiological assessments: Combining EEG with complementary methods such as functional near-infrared spectroscopy, EMG, heart rate variability, or respiratory monitoring may help characterize the dynamic interplay between cortical, muscular, and cardiorespiratory systems during fatigue development.
- Experimental modulation of brain activity: Real-time EEG-based interventions (e.g., neurofeedback, transcranial stimulation) could help determine whether altering specific oscillatory patterns, such as reducing Beta or enhancing Theta, is associated with changes in endurance performance or perceived exertion.
- Individual variability and adaptation: Investigating how factors such as training status, aerobic capacity, cognitive profile, or psychological resilience influence EEG–metabolic responses may inform the development of more personalized approaches for athletes with distinct performance profiles.
- Field-based EEG applications: The design of portable, motion-resistant EEG systems coupled with biofeedback capabilities may eventually allow exploratory monitoring and adjustment of effort pacing in naturalistic endurance environments.

Overall, advancing this line of research could contribute to the development of brain-centered training strategies, by leveraging cortical adaptations to better understand performance regulation, optimize fatigue resistance, and refine pacing strategies in both elite and recreational athletes.

## 5. Conclusions

This study indicates that endurance performance appears to be influenced not only by metabolic supply but also by neural mechanisms related to effort perception, motor coordination, and ventilatory regulation. By analyzing EEG–metabolic ratios, we identified consistent associations between cortical oscillations, ventilatory responses, and endurance performance. These associations suggest that neural processes may contribute to acute fatigue regulation, although alternative explanations linked to metabolic strain cannot be excluded.

More specifically, our results revealed a significant negative correlation between beta power and the time spent at $\dot{V}$O_2_max, consistent with the idea that increased neuromuscular and cognitive demands under fatigue are associated with greater cortical involvement. When expressed as a ratio, Beta/$\dot{V}$CO_2_ was also significantly associated with the time to reach $\dot{V}$O_2_max, suggesting that beta oscillations may primarily reflect neuromuscular compensation mechanisms rather than direct metabolic efficiency. In contrast, alpha and theta power did not show direct associations with endurance, but were positively correlated with performance when expressed relative to metabolic markers. Specifically, the Theta/$\dot{V}$O_2_ and Theta/$\dot{V}$CO_2_ ratios were correlated with time spent at $\dot{V}$O_2_max and TLIM duration, while the Alpha/$\dot{V}$O_2_ and Alpha/$\dot{V}$CO_2_ ratios were correlated with time to reach $\dot{V}$O_2_max and TLIM duration. These patterns are consistent with a potential role of these oscillations in supporting ventilatory coordination, cognitive-motor integration, and metabolic efficiency during high-intensity exercise.

These findings introduce EEG–metabolic ratios as exploratory indicators of endurance regulation, bridging cortical dynamics and systemic physiological constraints. By integrating neural and metabolic dimensions, this approach may provide a more comprehensive framework to examine how the brain contributes to performance regulation under high-intensity sustained effort. Nonetheless, the functional interpretation of alpha and theta bands in exercise settings must remain cautious, as their roles are partly extrapolated from cognitive neuroscience and may not directly map onto effort-related brain dynamics.

From an applied perspective, EEG-based monitoring may eventually help inform pacing strategies and effort regulation. Moreover, cognitive training and neurofeedback interventions should be considered exploratory tools that could be investigated for their potential to modulate fatigue resistance by influencing brain activity in real time.

Nonetheless, the present study faces several limitations, including the technical challenges of EEG acquisition during intense cycling and the need for broader validation of EEG–metabolic ratios across different endurance contexts. Future research should pursue longitudinal, multimodal, and interventional designs with larger samples to better elucidate the functional role of cortical activity in endurance performance.

## Figures and Tables

**Table 1:** Characteristics of the participants.

Subjects (*n* = 30)	Mean	SD	Min	Max
Age (years)	25.4	4.5	18.0	35.0
Height (cm)	180.4	6.3	170.0	193.0
Weight (kg)	73.5	9.6	59.0	102.0
Body Mass Index (kg/m^2^)	22.5	1.9	18.4	27.4
Training per week (hour)	8.3	5.3	3.0	25.0
$\dot{V}$O_2_max (mL·kg^−1^·min^−1^)	58.3	7.5	44.0	75.0

Abbreviations: SD = standard deviation; $\dot{V}$O_2_max = maximal oxygen uptake.

**Table 2:** Physiological characteristics of the participants assessed during the incremental test.

Subjects (*n* = 30)	Mean	SD	Min	Max
$\dot{V}$O_2_max (mL·kg^−1^·min^−1^)	58.3	7.5	44.0	75.0
Absolute MAP (W)	308.0	38.5	240.0	420.0
Relative MAP (W/kg)	4.2	0.7	2.6	5.6
HRmax (beats·min^−1^)	186.4	8.2	171	200
%HRmax	95.9	5.3	82.4	105.3
RER	1.2	0.1	1.1	1.3

Abbreviations: SD = standard deviation; $\dot{V}$O_2_max = maximal oxygen uptake; MAP = maximal aerobic power; RER = respiratory exchange ratio; %HRmax = HRmax/age-predicted HRmax × 100, where age-predicted HRmax = 210 − 0.65 × age [59].

**Table 3:** Endurance performance metrics.

Variable	Mean	SD	Min	Max
Time to exhaustion (s)	732.4	319.8	217.0	1600.0
Time to reach $\dot{V}$O_2_max (s)	290.5	179.6	75.0	917.0
Time spent at $\dot{V}$O_2_max (s)	441.9	258.9	96.0	1257.0
RER	1.1	0.1	1.0	1.3
RPE	17.4	1.4	15.0	20.0

Abbreviations: SD = standard deviation; $\dot{V}$O_2_max = maximal oxygen uptake; RER = respiratory exchange ratio; RPE = rating of perceived exertion.

**Table 4:** Spearman’s correlation of EEG Power Spectral Density slopes, time limits and RPE.

Variable	Theta	Alpha	Beta	TLIM Duration	Time to Reach $\dot{V}$O_2_max	Time Spent at $\dot{V}$O_2_max
Theta	1	0.460 (*p* = 0.011) [0.101, 0.714]	0.270 (*p* = 0.148) [−0.106, 0.579]	−0.051 (*p* = 0.787) [−0.404, 0.315]	0.032 (*p* = 0.867) [−0.332, 0.388]	−0.123 (*p* = 0.516) [−0.464, 0.250]
Alpha	0.460 (*p* = 0.011) [0.101, 0.714]	1	0.569 (*p* = 0.001) * [0.235, 0.783]	−0.176 (*p* = 0.352) [−0.506, 0.200]	0.281 (*p* = 0.132) [−0.095, 0.587]	−0.498 (*p* = 0.006) [−0.738, −0.146]
Beta	0.270 (*p* = 0.148) [−0.106, 0.579]	0.569 (*p* = 0.001) * [0.235, 0.783]	1	−0.385 (*p* = 0.036) [−0.662, −0.015]	0.001 (*p* = 0.996) [−0.359, 0.361]	−0.542 (*p* = 0.002) * [−0.766, −0.201]
Alpha/beta	0.239 (*p* = 0.203) [−0.138, 0.555]	0.441 (*p* = 0.015) [0.078, 0.701]	−0.253 (*p* = 0.177) [−0.566, 0.124]	0.220 (*p* = 0.243) [−0.157, 0.541]	0.410 (*p* = 0.025) [0.043, 0.680]	0.043 (*p* = 0.821) [−0.322, 0.397]
RPE	−0.055 (*p* = 0.774) [−0.407, 0.312]	0.314 (*p* = 0.091) [−0.061, 0.612]	0.559 (*p* = 0.002) * [0.222, 0.777]	−0.073 (*p* = 0.700) [−0.423, 0.295]	0.262 (*p* = 0.161) [−0.114, 0.573]	−0.208 (*p* = 0.270) [−0.531, 0.169]

Spearman’s rank correlation coefficients with *p*-values; 95% confidence intervals in brackets. Statistical significance was assessed using a Bonferroni correction for multiple comparisons, with a new significance level set at α = 0.0033 (*). Abbreviations: $\dot{V}$O_2_max = maximal oxygen uptake; TLIM = Time-to-exhaustion test; RPE = rating of perceived exertion.

**Table 5:** Spearman’s correlation of EEG–metabolic ratios slopes, RPE and time limits.

Variable	TLIM Duration	Time to Reach $\dot{V}$O_2_max	Time Spent at $\dot{V}$O_2_max	RPE
Theta/$\dot{V}$O_2_	0.488 (*p* = 0.007) [0.134, 0.732]	0.159 (*p* = 0.401) [−0.216, 0.493]	0.561 (*p* = 0.002) * [0.224, 0.778]	−0.155 (*p* = 0.411) [−0.490, 0.219]
Alpha/$\dot{V}$O_2_	0.527 (*p* = 0.003) [0.182, 0.757]	0.666 (*p* < 0.001) * [0.368, 0.840]	0.254 (*p* = 0.175) [−0.123, 0.567]	−0.095 (*p* = 0.616) [−0.441, 0.275]
Beta/$\dot{V}$O_2_	0.063 (*p* = 0.741) [−0.305, 0.414]	0.151 (*p* = 0.424) [−0.223, 0.487]	−0.119 (*p* = 0.528) [−0.461, 0.253]	0.444 (*p* = 0.015) [0.081, 0.702]
Theta/$\dot{V}$CO_2_	0.607 (*p* < 0.001) * [0.285, 0.805]	0.277 (*p* = 0.138) [−0.099, 0.584]	0.635 (*p* < 0.001) * [0.324, 0.822]	−0.123 (*p* = 0.515) [−0.464, 0.249]
Alpha/$\dot{V}$CO_2_	0.611 (*p* < 0.001) * [0.290, 0.808]	0.722 (*p* < 0.001) * [0.453, 0.871]	0.318 (*p* = 0.087) [−0.057, 0.615]	−0.048 (*p* = 0.800) [−0.402, 0.318]
Beta/$\dot{V}$CO_2_	0.506 (*p* = 0.005) [0.156, 0.744]	0.557 (*p* = 0.002) * [0.219, 0.775]	0.275 (*p* = 0.141) [−0.101, 0.583]	0.289 (*p* = 0.122) [−0.088, 0.593]

Spearman’s rank correlation coefficients with *p*-values; 95% confidence intervals in brackets. Statistical significance was assessed using a Bonferroni correction for multiple comparisons, with a new significance level set at α = 0.0021 (*). Abbreviations: $\dot{V}$O_2_max = maximal oxygen uptake; TLIM = Time-to-exhaustion test; $\dot{V}$O_2_ = oxygen uptake; $\dot{V}$CO_2_ = carbon dioxide production; RPE = rating of perceived exertion.

**Table 6:** Spearman’s correlations between EEG power spectral density, EEG–metabolic ratios, RPE and ventilatory variables ($\dot{V}$O_2_ and $\dot{V}$CO_2_).

Variables	$\dot{V}$O_2_	$\dot{V}$CO_2_
Theta	0.339 (*p* = 0.067) [−0.035, 0.630]	0.273 (*p* = 0.144) [−0.103, 0.582]
Alpha	0.512 (*p* = 0.004) [0.163, 0.747]	0.385 (*p* = 0.037) [0.015, 0.662]
Beta	0.677 (*p* < 0.001) * [0.384, 0.846]	0.557 (*p* = 0.002) * [0.219, 0.775]
Alpha/beta	−0.027 (*p* = 0.888) [−0.384, 0.337]	−0.076 (*p* = 0.688) [−0.425, 0.292]
Theta/$\dot{V}$O_2_	−0.413 (*p* = 0.024) [−0.681, −0.046]	−0.362 (*p* = 0.05) [−0.646, 0.010]
Alpha/$\dot{V}$O_2_	−0.208 (*p* = 0.269) [−0.532, 0.169]	−0.268 (*p* = 0.152) [−0.577, 0.109]
Beta/$\dot{V}$O_2_	0.124 (*p* = 0.511) [−0.248, 0.465]	0.078 (*p* = 0.681) [−0.291, 0.427]
Theta/$\dot{V}$CO_2_	−0.435 (*p* = 0.017) [−0.697, −0.071]	−0.471 (*p* = 0.009) [−0.720, −0.113]
Alpha/$\dot{V}$CO_2_	−0.217 (*p* = 0.248) [−0.538, 0.160]	−0.343 (*p* = 0.064) [−0.632, 0.031]
Beta/$\dot{V}$CO_2_	−0.222 (*p* = 0.238) [−0.542, 0.155]	−0.368 (*p* = 0.046) [−0.650, 0.004]
RPE	0.372 (*p* = 0.044) [0.001, 0.653]	0.246 (*p* = 0.189) [−0.131, 0.561]

Spearman’s rank correlation coefficients with *p*-values; 95% confidence intervals in brackets. Statistical significance was assessed using a Bonferroni correction for multiple comparisons, with a new significance level set at α = 0.0023 (*). Abbreviations: $\dot{V}$O_2_ = oxygen uptake; $\dot{V}$CO_2_ = carbon dioxide production; RPE = rating of perceived exertion.

**Table 7:** Simple regression models predicting endurance outcomes from EEG, EEG–metabolic, and ventilatory slopes.

Predictor	TLIM Duration (β (SE), R^2^)	*p*-Value	Time to Reach $\dot{V}$O_2_max (β (SE), R^2^)	*p*-Value	Time Spent at $\dot{V}$O_2_max (β (SE), R^2^)	*p*-Value
Theta	−0.039 (0.189), 0.005	0.837	0.107 (0.188), 0.011	0.575	−0.122 (0.188), 0.015	0.520
Alpha	−0.107 (0.188), 0.011	0.573	0.289 (0.181), 0.084	0.121	−0.333 (0.178), 0.111	0.072
Beta	−0.349 (0.177), 0.122	0.058	−0.092 (0.188), 0.008	0.631	−0.368 (0.176), 0.135	0.045
Theta/$\dot{V}$O_2_	0.350 (0.177), 0.123	0.058	0.225 (0.184), 0.051	0.232	0.277 (0.182), 0.076	0.139
Alpha/$\dot{V}$O_2_	0.363 (0.176), 0.132	0.049	0.472 (0.167), 0.222	0.009	0.121 (0.188), 0.015	0.525
Beta/$\dot{V}$O_2_	−0.037 (0.189), 0.001	0.847	0.125 (0.187), 0.016	0.510	−0.132 (0.187), 0.017	0.486
Theta/$\dot{V}$CO_2_	0.444 (0.169), 0.198	0.014	0.269 (0.182), 0.072	0.151	0.362 (0.176), 0.131	0.049
Alpha/$\dot{V}$CO_2_	0.401 (0.173), 0.161	0.028	0.449 (0.169), 0.202	0.013	0.183 (0.186), 0.033	0.333
Beta/$\dot{V}$CO_2_	0.451 (0.169), 0.203	0.012	0.371 (0.176), 0.137	0.044	0.299 (0.180), 0.089	0.108
$\dot{V}$O_2_	−0.546 (0.158), 0.298	0.002 *	−0.243 (0.183), 0.059	0.195	−0.505 (0.163), 0.255	0.004
$\dot{V}$CO_2_	−0.556 (0.157), 0.309	0.001 *	−0.303 (0.180), 0.092	0.104	−0.477 (0.166), 0.227	0.008

Values are standardized regression coefficients (β) with standard errors (SE). R^2^ values represent the variance explained by each univariate regression model. *p*-values are uncorrected; significance was evaluated against a Bonferroni-adjusted threshold (α = 0.0033). Asterisks (*) denote *p* < 0.0033. Abbreviations: $\dot{V}$O_2_ = oxygen uptake; $\dot{V}$CO_2_ = carbon dioxide production; $\dot{V}$O_2_max = maximal oxygen uptake; TLIM = Time-to-exhaustion test.

## Data Availability

Data presented in this study are available on request from the corresponding author. Data are not publicly available due to privacy.

## Supplementary Materials

The following supporting information can be downloaded at: https://www.mdpi.com/article/10.3390/jfmk10040369/s1, Figures S1–S3 illustrate the time course of EEG activity (Alpha, Beta), oxygen uptake ($\dot{V}$O_2_), heart rate, and EEG–metabolic ratios (e.g., Alpha/$\dot{V}$O_2_, Beta/$\dot{V}$CO_2_) during the TLIM90%MAP in a representative participant. All variables are expressed as percentages of their respective maximum values. Vertical dashed lines mark the attainment of $\dot{V}$O_2_max and the point of exhaustion. These visualizations are provided to illustrate the typical dynamic changes observed during the test.
