# Supplementary material for: EEG–Metabolic Coupling and Time Limit at V˙O2max During Constant-Load Exercise
Source: J Funct Morphol Kinesiol. 2025 Sep 26;10(4):369. doi: 10.3390/jfmk10040369 (PMC12550976; doi:10.3390/jfmk10040369)
Supplement: Supplementary file 1 [file jfmk-10-00369-s001.zip › jfmk-3884894-supplementary.pdf]

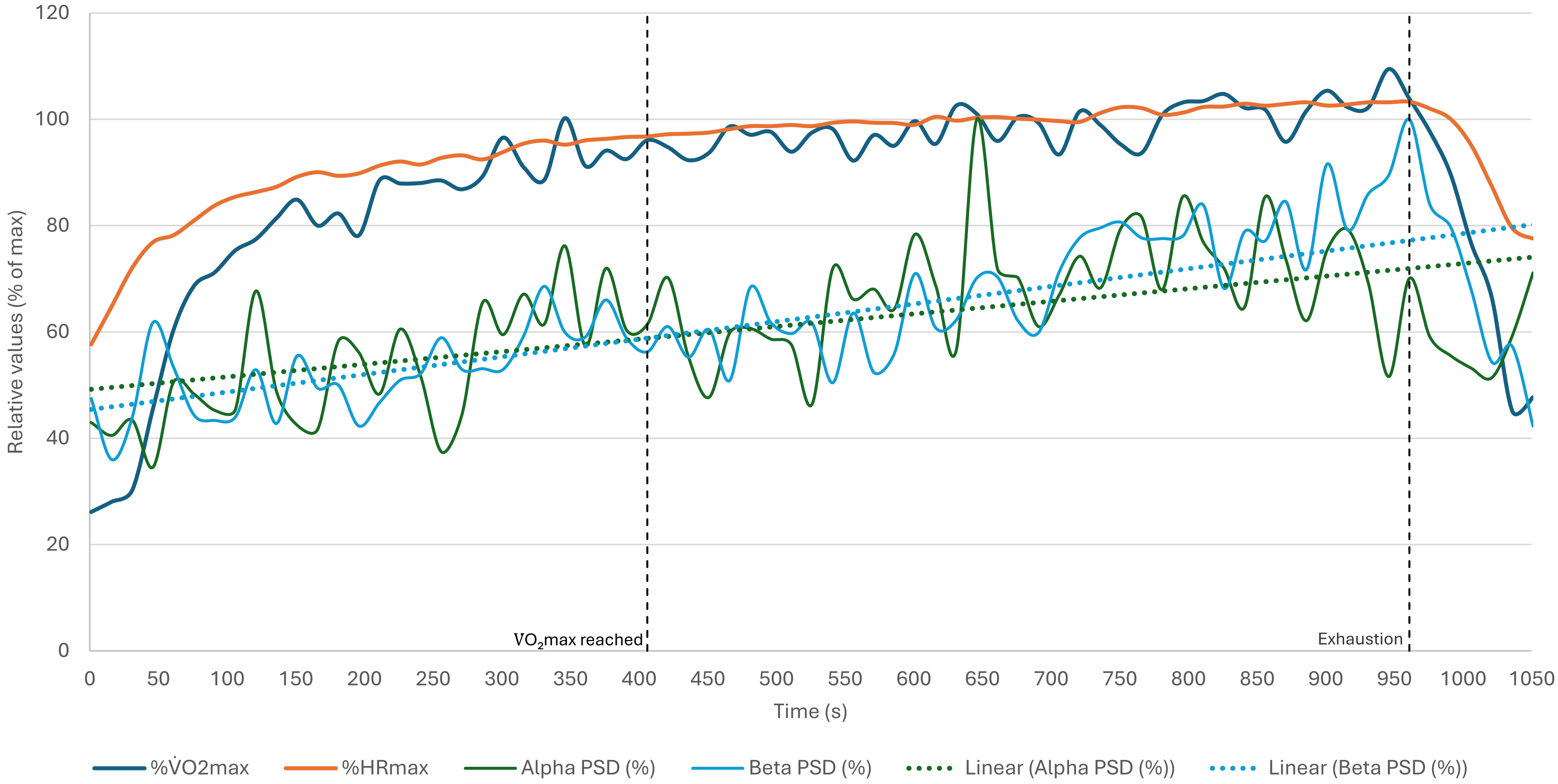

Figure S1. Dynamic changes in Alpha PSD, Beta PSD, $\dot{V}O_2$, and heart rate during the time-to-exhaustion test at 90% of maximal aerobic power in a representative subject. All variables are expressed as percentages of their individual maximal values. Dashed regression lines represent the overall trends in Alpha and Beta PSD evolution. The vertical dotted lines indicate the moment when $\dot{V}O_2$max was reached and the point of exhaustion.

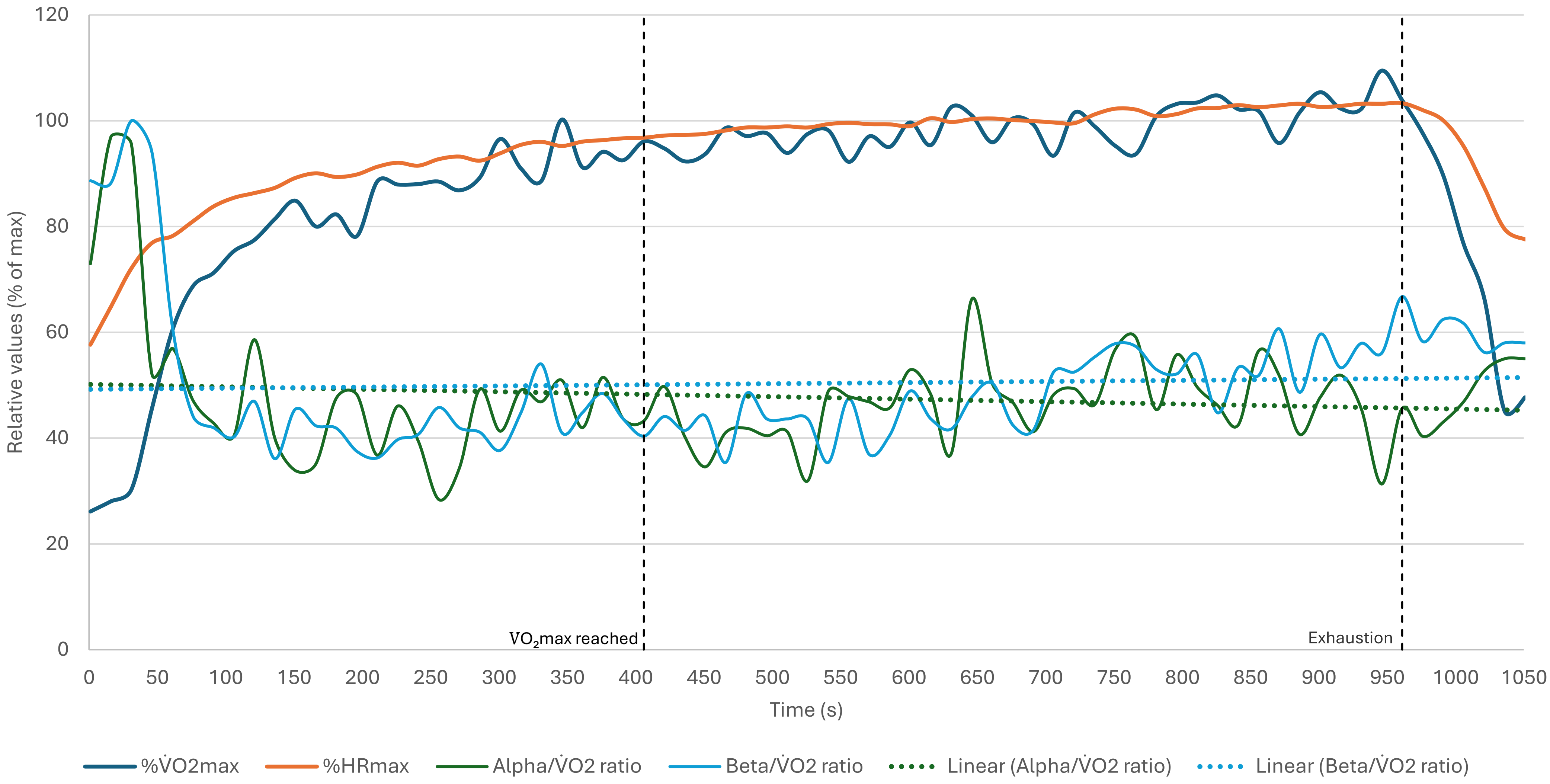

Figure S2. Dynamic changes in Alpha/$\dot{V}O_2$, Beta/$\dot{V}O_2$, $\dot{V}O_2$, and heart rate during the time-to-exhaustion test at 90% of maximal aerobic power in a representative subject. All variables are expressed as percentages of their individual maximal values. Dashed regression lines represent the overall trends in Alpha/$\dot{V}O_2$ and Beta/$\dot{V}O_2$ evolution. The vertical dotted lines indicate the moment when $\dot{V}O_2$max was reached and the point of exhaustion.

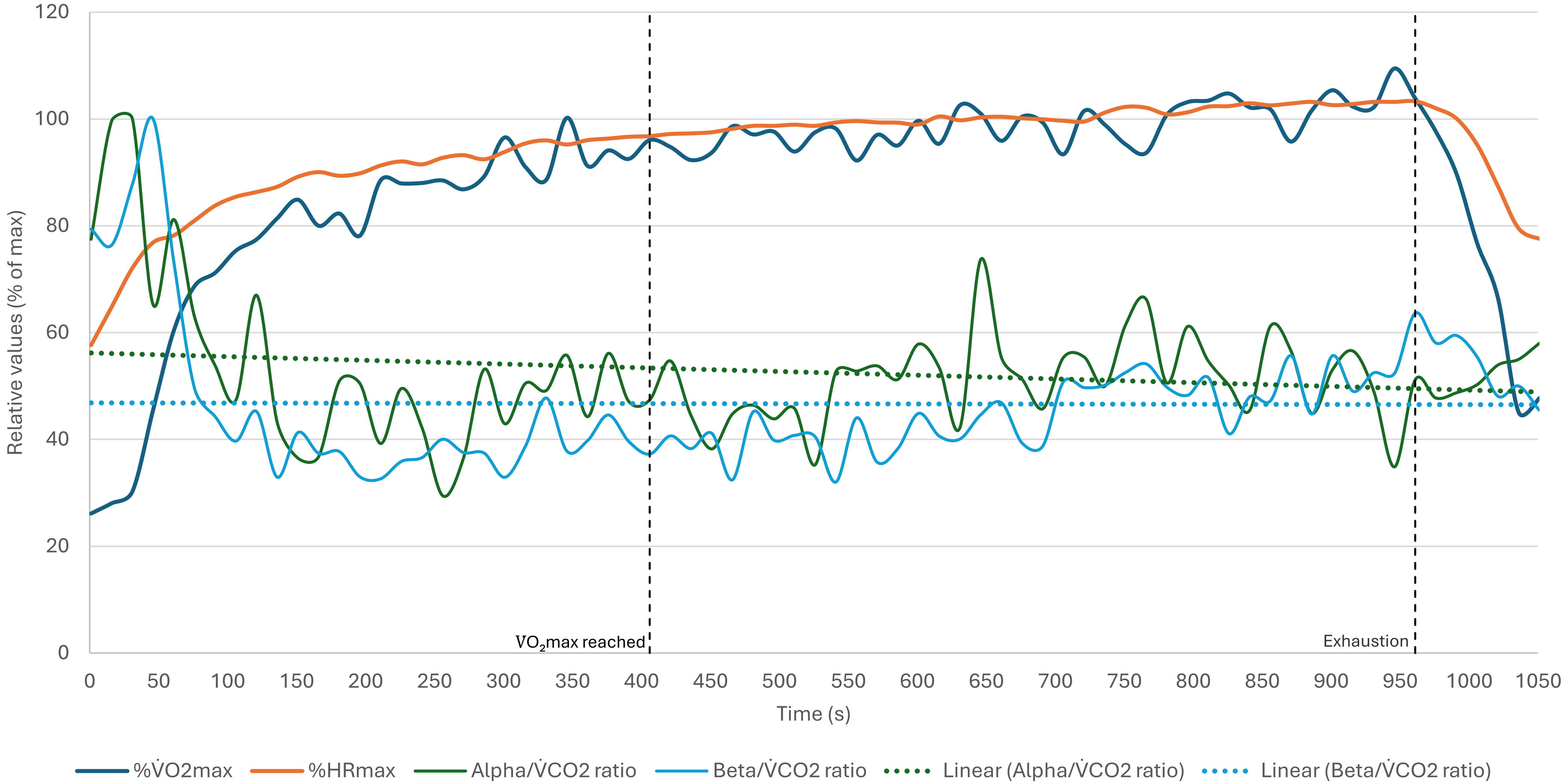

Figure S3. Dynamic changes in Alpha/$\dot{V}CO_2$, Beta/$\dot{V}CO_2$, $\dot{V}O_2$, and heart rate during the time-to-exhaustion test at 90% of maximal aerobic power in a representative subject. All variables are expressed as percentages of their individual maximal values. Dashed regression lines represent the overall trends in Alpha/$\dot{V}CO_2$ and Beta/$\dot{V}CO_2$ evolution. The vertical dotted lines indicate the moment when $\dot{V}O_2$max was reached and the point of exhaustion.
